# Cluster Survey Evaluation of a Measles Vaccination Campaign in Jharkhand, India, 2012

**DOI:** 10.1371/journal.pone.0127105

**Published:** 2015-05-26

**Authors:** Heather M. Scobie, Arindam Ray, Satyabrata Routray, Anindya Bose, Sunil Bahl, Stephen Sosler, Kathleen Wannemuehler, Rakesh Kumar, Pradeep Haldar, Abhijeet Anand

**Affiliations:** 1 Global Immunization Division, Centers for Disease Control and Prevention, Atlanta, Georgia, United States of America; 2 Epidemic Intelligence Service, Centers for Disease Control and Prevention, Atlanta, Georgia, United States of America; 3 National Polio Surveillance Project, World Health Organization Country Office, New Delhi, India; 4 Ministry of Health and Family Welfare, Government of India, New Delhi, India; London School of Hygiene and Tropical Medicine, UNITED KINGDOM

## Abstract

**Introduction:**

India was the last country in the world to implement a two-dose strategy for measles-containing vaccine (MCV) in 2010. As part of measles second-dose introduction, phased measles vaccination campaigns were conducted during 2010–2013, targeting 131 million children 9 months to <10 years of age. We performed a post-campaign coverage survey to estimate measles vaccination coverage in Jharkhand state.

**Methods:**

A multi-stage cluster survey was conducted 2 months after the phase 2 measles campaign occurred in 19 of 24 districts of Jharkhand during November 2011–March 2012. Vaccination status of children 9 months to <10 years of age was documented based on vaccination card or mother’s recall. Coverage estimates and 95% confidence intervals (95% CI) for 1,018 children were calculated using survey methods.

**Results:**

In the Jharkhand phase 2 campaign, MCV coverage among children aged 9 months to <10 years was 61.0% (95% CI: 54.4–67.7%). Significant differences in coverage were observed between rural (65.0%; 95% CI: 56.8–73.2%) and urban areas (45.6%; 95% CI: 37.3–53.9%). Campaign awareness among mothers was low (51.5%), and the most commonly reported reason for non-vaccination was being unaware of the campaign (69.4%). At the end of the campaign, 53.7% (95% CI: 46.5–60.9%) of children 12 months to <10 years of age received ≥2 MCV doses, while a large proportion of children remained under-vaccinated (34.0%, 95% CI: 28.0–40.0%) or unvaccinated (12.3%, 95% CI: 9.3–16.2%).

**Conclusions:**

Implementation of the national measles campaign was a significant achievement towards measles elimination in India. In Jharkhand, campaign performance was below the target coverage of ≥90% set by the Government of India, and challenges in disseminating campaign messages were identified. Efforts towards increasing two-dose MCV coverage are needed to achieve the recently adopted measles elimination goal in India and the South-East Asia region.

## Introduction

Measles caused an estimated 122,000 vaccine-preventable deaths worldwide in 2012 [1]. After implementing the World Health Organization’s (WHO) measles mortality reduction strategies, including delivery of two measles doses, estimated measles deaths declined by 78% globally, and by 63% in the South-East Asia Region (SEAR) during 2000–2012 [1,2]. In September 2013, the SEAR member countries, including India, became the last of the six WHO regions to adopt a regional goal of measles elimination.

India had a population of 1.2 billion in 2011, with 27 million annual live births [3,4]. The first dose of measles-containing vaccine (MCV1) given at 9–12 months of age was introduced into India’s Universal Immunization Programme (UIP) in 1985 [5]. In 2010, WHO and United Nations Children’s Fund (UNICEF) estimated India’s MCV1 coverage was 74% among children <12 months of age [6,7]. As a result, more than 10 million infants in India were estimated to be susceptible to measles in 2010 [5].

India was the last WHO country to implement a two-dose measles vaccination strategy [5]. During 2010–2011, a second MCV dose (MCV2) was introduced into India’s UIP through routine immunization (RI) services in 17 states with >80% MCV1 coverage among children aged 12–23 months in the District Level Household and Facility Survey conducted during 2007–2008 (DLHS-3); four other states with >80% MCV1 coverage had introduced a combined measles, mumps, and rubella vaccine before 2010 [5,8,9,10]. In the remaining 14 “priority states” with ≤80% MCV1 coverage, MCV2 introduction included a strategy of measles campaigns in three phases targeting 131 million children aged 9 months to <10 years during November 2010–November 2013, followed 6 months later by introduction into the RI program [11].

Jharkhand, with a population of 32,988,134 in the 2011 census, is a priority state where a measles campaign was conducted in two phases before MCV2 introduction into the RI program in October 2012 [5]. In Jharkhand, estimated MCV1 coverage by DLHS-3 was 71% among children aged 12–23 months [10]. During the phase 1 measles campaign, 721,578 children were vaccinated in 5 of 24 districts, and 5,377,566 children were vaccinated in the remaining 19 districts during phase 2 (Table 1) [11]. Districts included in the phases were based on operational feasibility and geographical contiguity and not based on variability in population or perceived immunity gaps. Campaign vaccination was administered at fixed sites, including regular RI and outreach sites, central urban sites, and schools, as well as by special teams targeting mobile and high-risk populations. A rapid coverage assessment was performed immediately after each phase [12].

**Table 1:** Districts covered in phase 1 and 2 measles vaccination campaigns, Jharkhand, 2010–2012.

Phase	District
1	Deoghar
	Gumla
	Jamtara
	Khunti
	Lohardaga
2	Bokaro
	Chatra
	Dhanbad
	Dumka
	Garhwa
	Giridih
	Godda
	Hazaribagh
	Koderma
	Latehar
	Pakur
	Palamu
	Ramgarh
	Ranchi
	Sahibganj
	Saraikella
	Simdega
	Singhbhum East
	Singhbhum West

We conducted a household-level cluster survey following the phase 2 measles campaign in Jharkhand during November 2011–March 2012 to evaluate vaccination coverage of children aged 9 months to <10 years and living in the 19 targeted districts. Our evaluation provides overall estimates of measles campaign coverage for Jharkhand state and by rural and urban areas; identifies risk factors and reported reasons for non-vaccination during the campaign; assesses the effectiveness of the communication strategy in promoting campaign awareness; and estimates the proportions of children fully vaccinated (≥2 MCV doses), under-vaccinated (1 MCV dose), or unvaccinated against measles after the campaign.

## Methods

### Ethics Statement

This evaluation was determined not to be human subjects research and approved by the Office of the Associate Director for Science, Center for Global Health, U.S. Centers for Disease Control and Prevention (CDC) as a public health program evaluation activity. Because the evaluation was not human subjects research, a consent procedure was approved where verbal consent was obtained from participants at the beginning of the survey as part of a standard script, and refusal was documented on forms by survey teams.

### Survey methodology

In May 2012, a multi-stage cluster survey was conducted to estimate measles vaccine coverage in rural and urban areas of Jharkhand state after the phase 2 measles campaign conducted during November 2011–March 2012. The urban sampling frame of primary sampling units (clusters) was from the 2011 Urban Frame Survey of the GOI National Sample Survey Office (NSSO); the rural sampling frame was from the 2004 Census of India block listing with urban blocks in the urban sampling frame subtracted. Both were limited to the 19 targeted districts.

The target sample size was 400 children each in the urban and rural surveys, which was calculated based on an estimated 50% vaccination coverage, desired precision of +/- 7.5%, 95% probability of achieving that precision, design effect of 2, and 15% nonresponse rate. Each survey consisted of 40 clusters, sampled systematic probability proportional to estimated size. It was estimated that 75% of urban and 99% of rural households would have at least one child in the target age group. We calculated the number of sampled households (trials) needed to insure 10 eligible households were identified 90% of the time, assuming a binomial distribution with probability equal to the proportion of households having an eligible child. We initially determined that sampling 19 households in urban clusters and 13 in rural would insure that 10 eligible households would be identified in each cluster with probability >0.95. This was further inflated to 22 urban and 18 rural households out of concern that the estimated proportion of households with an eligible child was an over-estimate. Households were defined as persons living together and eating from the same kitchen.

Survey teams were comprised of trained volunteers of the National Polio Surveillance Project (NPSP), WHO Country Office for India. Survey teams used maps of urban clusters from NSSO and polio microplan maps of rural clusters from NPSP. Clusters larger than 400 households were segmented into quadrants, and a quadrant was randomly selected. Clusters were not enumerated; therefore, the skip interval used to select households was determined by dividing the estimated number of households in the cluster by the desired number of households. The first household and subsequent households were selected by methods previously described [13]. In households with more than one mother with eligible children, a Kish grid was used to randomly select one [14]. After obtaining verbal consent, a standard paper questionnaire was completed for the selected mother (or caretaker), including socio-demographic information and the vaccination status of all her eligible children aged 9 months to <10 years and living in Jharkhand at the start of the campaign. Measles vaccination status was assessed based on vaccination card or mother’s recall. Teams were monitored daily by Surveillance Medical Officers (SMOs) and national level NPSP staff using monitoring forms to assess data quality and adherence to survey protocol; data collected in one cluster was of insufficient quality and required revisiting. Survey teams and supervisors successfully visited all sampled clusters, including a forest village in an insecure zone requiring 7 km trek by foot. Data were entered into Microsoft Access and cleaned by the NPSP data team.

### Analysis method

During analysis, one eligible child was selected by simple random sampling from each household to limit intra-household correlation. Sampling weights accounted for the first stage selection, the estimated second stage selection including the segmentation, and the known selection probability of one mother and one child per household. Weights were then post-stratified to account for differences in the sex ratios of our urban and rural samples compared with the sex ratios reported in the 2011 census. To determine relative wealth quintiles, principal component analysis was performed separately for the urban and rural surveys to calculate a wealth index based on household indicators as described [15]. Indicators included utilities and characteristics of the household dwelling, persons per dwelling room, land ownership, and ownership of consumer durable goods. Households were placed in wealth quintiles based on their index score relative to the population in their respective survey. Coverage estimates and 95% confidence intervals (CI) were calculated separately for each survey (urban and rural) using Taylor series linearization method to account for survey design. We report the Wilson CIs for estimates ≤20% and ≥80%, and the Wald CI using the t-distribution for all others. Comparisons in coverage estimates between socio-demographic sub-populations were made using the Rao-Scott chi-square test (p-value of <0.05 assigned statistical significance). Design effect (DE) and intraclass correlation coefficients (ICCs) were calculated for the urban/rural surveys. Organ-pipe plots were constructed by plotting unweighted coverage by cluster (Dale Rhoda, personal communication). Descriptive analysis was also conducted for other measles campaign and RI data, including reasons for non-vaccination and sources of campaign information. Measles vaccination status was missing for 13 campaign responses and 18 RI responses. All analyses were conducted using SAS version 9.3.

## Results

### Characteristics of the survey sample

Of the 19 districts included in the phase 2 measles campaign, selected rural and urban clusters were in 19 and 12 districts, respectively. Overall, 1,581 houses were visited (872 in urban and 709 in rural), and 1,022 households (64.6%) were surveyed. Of the 1,581 houses visited, 513 (32.4%) had no eligible children, 42 (2.7%) had no one home after two attempts, and 4 (0.3%) refused participation. Of the 1,022 households surveyed, 79 (7.7%) had more than one mother with eligible children, and 530 (51.9%) had more than one eligible child. After excluding ineligible children and selecting one eligible child per household, 1,018 children (504 from urban areas and 514 from rural areas) were included in the analysis.

Of the 504 urban and 514 rural households surveyed, the majority of household heads in urban (62.7%) and rural (90.7%) areas self-identified as scheduled tribe, scheduled caste, or other backward class (Table 2). Median household size was six persons for urban (range: 1–26) and rural areas (range: 1–30). Urban households more often reported having homes with finished floors (71.8%) and electricity (93.5%) and less often reported open defecation (56.2%) than rural households (23.9%, 48.2%, and 90.9%). Rates of literacy were higher among urban mothers (68.8%) and their husbands (83.0%) than rural parents (35.2% and 65.4%). Male children represented 54.6% of the urban sample and 49.8% of the rural sample. Children aged 5 to <10 years comprised 54.6% of the urban sample and 53.5% of the rural sample; most children in this age group attended school (urban: 91.6%; rural: 90.9%). Children aged 9 months to <5 years of age comprised 45.4% and 46.5% of the urban and rural samples, respectively; a large proportion of children in this age group were also reported to attend school (urban: 43.7%; rural: 46.5%) (Table 2).

**Table 2:** Socio-demographic characteristics of children in the urban and rural surveys, Jharkhand, 2012.

	Urban		Rural		Total	
Characteristic	No.	%	No.	%	No.	%
Sex						
Male	275	54.6	256	49.8	531	52.2
Female	228	45.2	258	50.2	486	47.7
Missing	1	0.2	-	-	1	0.1
Age group						
5 to <10y	275	54.6	275	53.5	550	54.0
9m to <5y	229	45.4	239	46.5	468	46.0
Child's school attendance						
5 to <10y, in school	252	50.0	250	48.6	502	49.3
5 to <10y, not in school	22	4.4	25	4.9	47	4.6
9m to <5y, in school	100	19.8	62	12.1	162	15.9
9m to <5y, not in school	128	25.4	173	33.7	301	29.6
Missing	2	0.4	4	0.8	6	0.6
Scheduled caste, tribe or other backward class						
General	184	36.5	45	8.8	229	22.5
Other backward class	175	34.7	243	47.3	418	41.1
Scheduled tribe	78	15.5	169	32.9	247	24.3
Scheduled caste	63	12.5	54	10.5	117	11.5
Missing	4	0.8	3	0.6	7	0.7
Religion						
Hindu	376	74.6	347	67.5	723	71.0
Muslim	66	13.1	74	14.4	140	13.8
Other	61	12.1	93	18.1	154	15.1
Missing	1	0.2	-	-	1	0.1
Household owns home						
Own	283	56.2	501	97.5	784	77.0
Rent	120	23.8	4	0.8	124	12.2
Informal settlement	97	19.2	1	0.2	98	9.6
Missing	4	0.8	8	1.6	12	1.2
Household size						
≤6 persons	323	64.1	295	57.4	618	60.7
>6 persons	181	35.9	218	42.4	399	39.2
Missing	-	-	1	0.2	1	0.1
Mother's no. of children aged 9m to <10y						
<2 children	257	51.0	233	45.3	490	48.1
≥2 children	247	49.0	281	54.7	528	51.9
Mother's school completion						
Middle school or higher	276	54.8	84	16.3	360	35.4
None or Primary	214	42.5	369	71.8	583	57.3
Missing	14	2.8	61	11.9	75	7.4
Mother's literacy						
Literate	347	68.8	181	35.2	528	51.9
Illiterate	150	29.8	328	63.8	478	47.0
Missing	7	1.4	5	1.0	12	1.2
Husband's school completion						
Middle school or higher	341	67.7	179	34.8	520	51.1
None or primary	148	29.4	299	58.2	447	43.9
Missing	15	3.0	36	7.0	51	5.0
Husband's literacy						
Literate	419	83.1	336	65.4	755	74.2
Illiterate	78	15.5	175	34.0	253	24.9
Missing	7	1.4	3	0.6	10	1.0
Husband's type of work						
Skilled or professional	122	24.2	81	15.8	203	19.9
Farmer	10	2.0	156	30.4	166	16.3
Unskilled	323	64.1	226	44.0	549	53.9
Other	24	4.8	25	4.9	49	4.8
Missing	25	5.0	26	5.1	51	5.0

Abbreviations: y = years, m = months

### Campaign measles vaccination coverage

**Fig 1:**
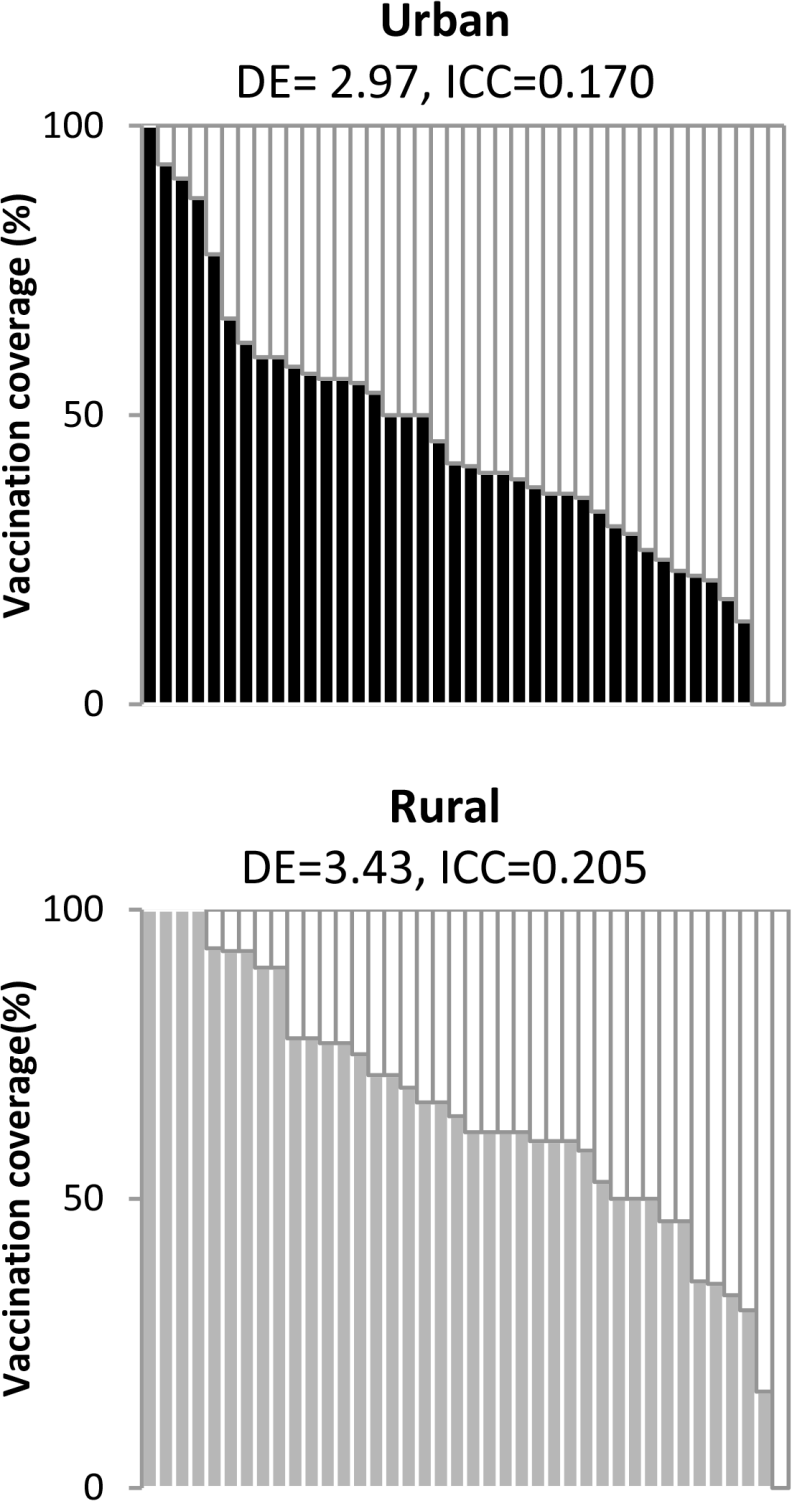
Cluster-level measles campaign vaccination coverage in the urban and rural surveys, Jharkhand, 2012. Organ-pipe plots of unweighted coverage by cluster were constructed for urban and rural surveys (Dale Rhoda, personal communication). Design effect (DE) and intraclass correlation coefficients (ICCs) were calculated for the respective surveys.

An estimated 61.0% (95% CI: 54.4%–67.7%) of the children in the 19 districts of Jharkhand received measles vaccine in the phase 2 measles campaign, as assessed by vaccination card or mother’s recall. Campaign vaccination card availability was low (25.1%). Coverage was significantly higher (p = 0.001) in rural areas (65.0%; 95% CI: 56.8%–73.2%) than in urban areas (45.6%; 95% CI: 37.3%–53.9%). Cluster-level coverage varied substantially (range: 0‒100%) in both urban and rural areas, with 0% coverage among children surveyed in one rural and two urban clusters (Fig 1). No significant differences in coverage by sex were observed in either the urban or rural areas, or by age and school attendance in urban areas. In rural areas, coverage differed by age (p = 0.033), child’s school attendance (p = 0.025), and religion of the household (p = 0.023). Among rural children in school, coverage was similar in both age groups (72.3% vs. 70.8%). Coverage was 38.1% (95% CI: 19.7%–56.6%) among rural children with “other religion,” which included 60% Sarna and 34% Christian (Table 3).

**Table 3:** Phase 2 measles vaccination campaign coverage estimates by socio-demographic characteristic for urban and rural areas, Jharkhand, 2012.

	Urban						Rural					
Characteristic	%	LCL	UCL	Vacci-nated no.	Total no.^a^	P-value^a^	%	LCL	UCL	Vacci-nated no.	Total no.^a^	P-value^b^
Overall	45.6	37.3	53.9	227	495	-	65.0	56.8	73.2	327	509	0.001* ^c^
Sex												
Male	49.9	41.4	58.4	130	270	0.080	62.6	53.3	72.0	157	252	0.393
Female	41.0	30.4	51.7	97	225		67.4	57.1	77.8	170	257	
Age group												
5 to <10y	45.4	36.4	54.4	126	269	0.920	71.4	63.3	79.4	192	274	0.033*
9m to <5y	45.9	35.0	56.8	101	226		57.0	44.3	69.7	135	235	
Child's school attendance												
5 to <10y, in school	46.8	37.2	56.5	120	247	0.501	72.3	64.1	80.5	178	249	0.025*
5 to <10y, not in school	30.8	11.7	49.9	6	22		62.2	40.7	83.7	14	25	
9m to <5y, in school	50.3	36.3	64.4	50	99		70.8	51.8	89.8	41	61	
9m to <5y, not in school	43.3	29.3	57.4	51	126		53.0	39.7	66.3	94	170	
Scheduled caste, tribe or other backward class												
General	40.9	30.3	51.5	73	178	0.102	63.5	46.1	80.8	26	45	0.180
Other backward class	55.2	41.7	68.6	96	173		69.9	59.9	79.9	167	239	
Scheduled tribe	45.9	31.0	60.9	35	78		56.3	42.6	70.0	94	168	
Scheduled caste	31.7	16.0	47.4	21	62		73.5	57.7	89.2	40	54	
Religion												
Hindu	45.7	36.5	54.9	165	370	0.513	72.2	64.8	79.7	245	344	0.023*
Muslim	37.5	6.1	68.9	30	63		64.8	49.7	79.9	46	72	
Other	56.8	43.7	70.0	32	61		38.1	19.7	56.6	36	93	
Household size												
≤6 persons	43.5	35.0	52.1	140	318	0.447	65.5	56.2	74.8	192	294	0.803
>6 persons	48.5	35.9	61.0	87	177		64.4	55.2	73.7	134	214	
Mother's no. of children aged 9m to <10y															
<2 children	41.2	32.7	49.6	108	253	0.151	65.1	56.2	73.9	151	232	0.986
≥2 children	47.6	38.0	57.3	119	242		65.0	56.2	73.8	176	277	
Mother's school completion												
Middle school or higher	46.3	35.5	57.1	123	268	0.810	67.0	50.5	83.6	54	82	0.967
None or Primary	44.8	34.3	55.2	97	213		66.7	57.7	75.7	239	366	
Mother's literacy												
Literate	48.9	39.8	58.0	165	338	0.072	67.3	56.3	78.2	112	179	0.561
Illiterate	38.2	26.9	49.6	59	150		63.9	54.6	73.2	212	325	
Husband's school completion												
Middle school or higher	45.2	36.1	54.3	152	333	0.651	69.9	59.1	80.7	119	176	0.206
None or primary	48.1	35.4	60.8	71	147		62.8	53.0	72.6	185	297	
Husband's literacy												
Literate	46.3	37.5	55.0	193	411	0.625	66.2	57.9	74.4	218	333	0.595
Illiterate	43.3	30.3	56.2	32	77		63.1	50.5	75.6	107	173	
Husband's type of work												
Skilled or professional	42.9	30.8	55.0	56	119	0.576	70.5	60.0	81.0	54	81	0.618
Farmer	-	-	-	-	-		62.1	45.5	78.7	95	155	
Unskilled	45.0	36.4	53.6	144	317		63.1	51.7	74.5	142	224	
Other	54.6	32.5	76.7	16	34		74.3	59.4	89.1	16	24	
Household owns home												
Own	52.5	42.0	63.0	143	277	0.097	-	-	-	-	-	-
Rent	34.6	24.9	44.3	44	117		-	-	-	-	-	
Informal settlement	38.5	20.1	56.9	38	97		-	-	-	-	-	
Relative wealth quintile												
1	42.9	27.9	58.0	41	99	0.139	62.5	47.6	77.4	57	98	0.883
2	48.1	32.7	63.4	45	99		70.2	58.1	82.4	70	100	
3	56.0	40.6	71.4	61	99		70.1	57.7	82.4	69	100	
4	49.6	38.5	60.7	47	93		65.3	51.5	79.0	63	98	
5	31.0	18.2	43.8	30	97		66.5	52.0	81.1	65	98	

Abbreviations: LCL = lower confidence limit, UCL = upper confidence limit, y = years, m = months

^a^ Variations in denominator due to missing data

^b^ Rao-Scott chi-square test; p-values denoted with * are statistically significant

^c^ P-value is for comparison between urban and rural surveys

### Reasons children were missed during the campaign

Among children not receiving the campaign measles vaccine, the primary reason reported by mothers in urban (74.3%) and rural areas (62.2%) was being unaware of the campaign. In urban areas, other reported reasons included absence of the child during the campaign (4.9%), lack of awareness of the need of vaccination (4.1%), and fear of pain from injection (3.7%). In rural areas, other reported reasons included the vaccination team not coming to the area (7.2%), lack of awareness of the need for vaccination (6.7%), and absence of the child during the campaign (6.1%) (Table 4). In the two urban clusters with 0% coverage, the majority (17/24) of reasons cited were being unaware of the campaign; in the one rural cluster with 0% coverage, the majority (11/15) of reasons cited were the vaccination team not coming to the appointed site. A portion (7.3%) of mothers reported that their child had a side effect after campaign vaccination; signs and symptoms reported were fever (6.0%), pain at the injection site (0.6%), and vomiting (0.2%).

**Table 4:** Reasons for non-vaccination and awareness of the phase 2 measles vaccination campaign by urban and rural areas, Jharkhand, 2012.

	Urban		Rural		Total	
Characteristic	No.	%	No.	%	No.	%
Reason child not vaccinated during campaign						
Unaware of campaign	199	74.3	112	62.2	311	69.4
Child absent or traveling	13	4.9	11	6.1	24	5.4
Unaware of need for vaccination	11	4.1	12	6.7	23	5.1
Fear of pain from injection	10	3.7	4	2.2	14	3.1
Vaccination team did not come	-	-	13	7.2	13	2.9
Fear of side effects of vaccination	5	1.9	5	2.8	10	2.2
Child sick, mother unwilling to let vaccinate	4	1.5	5	2.8	9	2.0
Child sick, health worker unwilling to vaccinate	2	0.7	4	2.2	6	1.3
Aware of campaign, but didn't know where to go	1	0.4	4	2.2	5	1.1
Aware of campaign, but unaware of session time	3	1.1	2	1.1	5	1.1
Vaccinator's behavior not friendly	2	0.7	2	1.1	4	0.9
Child already vaccinated	3	1.1	-	-	3	0.7
No faith in vaccination	2	0.7	1	0.6	3	0.7
Inconvenient session timing	1	0.4	-	-	1	0.2
Long waiting time	1	0.4	-	-	1	0.2
Vaccination team ran out of vaccine	1	0.4	-	-	1	0.2
Other	10	3.7	5	2.8	15	3.3
Mother had knowledge of the campaign						
Yes	228	45.2	296	57.6	524	51.5
No	273	54.2	216	42.0	489	48.0
Source of campaign knowledge						
Local health worker (ASHA/AWW/other)	92	40.4	216	73.0	308	58.8
School	106	46.5	49	16.6	155	29.6
ANM	10	4.4	18	6.1	28	5.3
Neighbor	6	2.6	5	1.7	11	2.1
Don't know	2	0.9	5	1.7	7	1.3
TV	2	0.9	1	0.3	3	0.6
Newspaper	3	1.3	-	-	3	0.6
Community leader/village head	1	0.4	-	-	1	0.2
Microphone announcement/miking	1	0.4	-	-	1	0.2
Poster/banner	-	-	1	0.3	1	0.2
Temple/church/mosque	1	0.4	-	-	1	0.2
Other	3	1.3	1	0.3	4	0.8

Abbreviations: ASHA = accredited social health activist, AWW = anganwadi workers, ANM = auxiliary nurse midwife

### Campaign awareness and sources of information

Higher coverage was observed among children whose mothers had knowledge of the campaign versus those who lacked knowledge of the campaign in both urban (77.1% versus 17.2%, p <0.001) and rural areas (87.8% versus 30.6%, p <0.001). Roughly half of mothers in urban (45.2%) and rural areas (57.6%) reported being aware of the measles campaign. Among those aware, the majority of mothers in urban areas learned about the campaign from school staff (46.5%) or a local health worker (40.4%). Most mothers in rural areas learned about the campaign from a local health worker (73.0%), followed by school staff (16.6%). Less than 2.0% of mothers reported hearing about the campaign from mass media, such as TV, newspaper, radio, posters, or microphone announcement (Table 4). Only 42.5% and 57.4% of mothers with vaccinated children in urban areas and rural areas, respectively, reported being told about RI services by vaccinators during the campaign.

### Routine measles vaccination

Higher campaign coverage was observed among children aged 12 months to <5 years who had previously received MCV1 through RI services compared with those unvaccinated in both urban (50.1% versus 26.7%, p = 0.003) and rural areas (69.8% versus 49.5%, p = 0.002). Among children aged 12 months to <5 years, overall estimated MCV1 coverage through RI services was 81.1% (95% CI: 73.9%–86.7%), by vaccination card or mother’s recall. No difference in MCV1 coverage existed in urban (80.9%; 95% CI: 70.0%–85.5%) and rural areas (81.2%; 95% CI: 72.4%–87.6%). Estimated MCV2 coverage through RI services among children aged 12 months to <5 years was 7.3% (95% CI: 3.4%–15.0%), by vaccination card or mother’s recall. RI card availability was low (32.3%).

Children were most commonly vaccinated with MCV1 at outreach government sites (urban: 44.3%, rural: 84.8%), followed by fixed government sites (urban: 31.4%, rural: 10.7%), and private providers (urban: 23.4%, rural: 2.9%). Among children not vaccinated with MCV1, the most commonly reported reason in urban (52.5%) and rural areas (37.3%) was lack of awareness for the need for vaccination. In urban areas, other reported reasons for non-vaccination were lack of awareness of the day/time for vaccination (6.3%), mother’s unwillingness to vaccinate because of child’s illness (6.3%), and fear of side effects (5.0%). In rural areas, other reported reasons for non-vaccination were lack of awareness of the place for vaccination (13.5%), lack of awareness of the day/time for vaccination (11.9%), and fear of side effects (10.3%).

### Increase in measles vaccination coverage due to the campaign

During the phase 2 measles campaign in Jharkhand, an estimated 6.3% (95% CI: 3.7%–10.4%) of children aged 12 months to <5 years and 13.3% (95% CI: 9.3%–18.6%) of children aged 5 to <10 years received their first MCV dose. The estimated proportion receiving their second MCV dose during the campaign was 44.4% (95% CI: 34.3%–54.4%) for children aged 12 months to <5 years and 49.8% (95% CI: 41.2%–58.3%) for children aged 5 to <10 years. At the end of the phase 2 campaign, an estimated 53.7% (95% CI: 46.5%–60.9%) of children aged 12 months to <10 years had received ≥2 MCV doses; an estimated 34.0% (95% CI: 28.0%–40.0%) had received one MCV dose; and 12.3% (95% CI: 9.3%–16.2%) remained unvaccinated (Table 5).

**Table 5:** Estimated proportion of children by age group and number of measles doses received at the end of the phase 2 measles vaccination campaign, Jharkhand, 2012.

		12m to <5y (n = 431)^a^				5 to <10y (n = 531)				12m to <10y (n = 962) ^a^			
Doses	Dose type	%	LCL	UCL	No.	%	LCL	UCL	No.	%	LCL	UCL	No.
0 doses	-	13.0	8.7	19.1	55	11.8	8.2	16.6	65	12.3	9.3	16.2	120
1 dose	1 RI dose	29.7	21.7	37.6	135	19.2	14.7	24.8	137	23.7	18.8	28.7	272
	1 campaign dose	6.3	3.7	10.4	22	13.3	9.3	18.6	58	10.3	7.3	14.2	80
2 doses	2 RI doses	3.1	0.9	10.5	19	2.7	0.8	8.7	17	2.9	0.8	9.4	36
	1 RI dose and 1 campaign dose	44.4	34.3	54.4	180	49.8	41.2	58.3	222	47.4	39.7	55.1	402
3 doses	2 RI doses and 1 campaign dose	3.5	1.4	8.8	19	3.2	1.4	7.2	32	3.4	1.4	7.7	51

Abbreviations: m = months, y = years, LCL = lower confidence limit, UCL = upper confidence limit, RI = routine immunization

^a^ Children aged 9‒11 months were excluded from this analysis, based on the RI vaccination schedule

## Discussion

The phase 2 measles campaign in Jharkhand achieved an overall coverage of 60.1% among children aged 9 months to <10 years. Although implementation of the campaign was a significant achievement towards measles elimination, campaign performance was below the target coverage of ≥90% set by the GOI [12]. For measles elimination, WHO recommends achieving and maintaining ≥95% coverage with two MCV doses in every district [16]. At the end of the phase 2 measles campaign in Jharkhand, just over half of children aged 12 months to <10 years were estimated to be fully vaccinated, while a large proportion remained under-vaccinated (34.0%) or unvaccinated (12.3%) against measles.

Reported administrative coverage, i.e. the proportion of children vaccinated among those targeted, for the phase 2 measles campaign in Jharkhand was 91.6%—higher than the 61.0% coverage estimated by this survey [11]. Administrative coverage is commonly used to asses campaign coverage, but is often unreliable [17,18]. Because of the noted discrepancy in administrative and survey coverage, future measles campaigns could include a post-campaign coverage survey to reliably estimate vaccination coverage.

Campaign coverage was much higher in rural (65.0%) versus urban areas (45.6%). This suggests that, in addition to improving overall coverage, special strategies for overcoming challenges specific to urban areas should be developed and deployed in future campaigns. Coverage was higher among those who had been previously vaccinated through RI services. This suggests that more effort is needed to identify geographic areas and sub-populations missed by RI services and to tailor strategies for use in future campaigns. From survey results, special attention may be required to reach children who are not in school, or have religions other than Hinduism and Islam.

Estimated MCV1 coverage through RI services among children aged 12 months to <5 years (81.1%) was higher than campaign coverage, and there was no disparity in coverage between urban and rural areas. This highlights the potential benefit that MCV2 introduction through RI services and further improvements in RI coverage might have on two-dose MCV coverage.

Only 51.5% of mothers reported awareness of the campaign, and the primary reason reported for non-vaccination was lack of awareness of the campaign. Most mothers reported hearing about the campaign through interpersonal communication (IPC), including local health workers and school staff, with mass media rarely being mentioned as a source of information. Additionally, only 51.3% of mothers reported being told about RI services during the campaign, and lack of awareness for the need for vaccination was the most commonly reported reason for not receiving MCV1 though RI services. Together, these findings underscore the need to improve the efficacy of messaging regarding RI services and future measles campaigns. In future campaigns, a timely, well-planned communication strategy that uses both IPC and mass media, and promotes the importance and value of immunization, will be required to achieve the desired coverage [19,20].

The survey analyses had limitations. Campaign vaccination card retention rates were low, and the use of mother’s recall to document vaccination status is less reliable [21]. Estimation of the total number of MCV doses received included MCV1 vaccination status for children aged 5 to <10 years as determined by mother’s recall, which is less reliable because of the time elapsed since vaccination. Though our analyses included sampling weights, the weights were calculated based on estimated second stage sampling probabilities, which may lead to bias in the estimated percentages. Bias may also have resulted if the sampled households were not representative of the entire cluster. The magnitude of the bias cannot be ascertained, but it is unlikely to affect the interpretation that campaign coverage was suboptimal. Finally, the results are not generalizable to other phases and geographical locations of measles campaigns in India.

To achieve the measles elimination in India, much higher MCV coverage will be required, whether delivered through RI and/or future campaigns. Because MCV2 has been introduced into RI services in all states in India, focusing efforts on achieving high measles two-dose coverage through RI services is recommended. Where campaigns are required, an emphasis should be placed on effectively disseminating campaign messages and reaching those previously unreached through RI services to achieve high coverage, similar to what has been done successfully for polio eradication in India [22]. Campaigns should also be used to spread information about RI services, and reinforce RI services through micro-planning, training, supervision, and improvements to cold chain [23,24]. Increased efforts to reach high two-dose MCV coverage though RI and campaigns in India will aid in achieving the SEAR measles elimination goal by 2020.
